# Extract of a Letter from Dr. De Carro to Dr. Jenner, Dated Vienna, Feb. 14, 1801
*We understand that this learned Physician is about to publish, in French and German, an elaborate History of this Inoculation, dedicated to Lord Minto, as Minister of the nation where the discovery took place.


**Published:** 1801-04

**Authors:** 


### *Extract of a Letter from* Dr. De Carro* *to* Dr. Jenner, dated Vienna, Feb. 14, 1801.

In the neighbourhood of Vienna I had lately an intereſting proof of the philanthropy of a clergyman, and of the good which that claſs of men can do, when they chuſe to do it. I had inoculated four children of a gentleman living in the village Brunnam Gebizg: the reſpectable curate of that pariſh, made daily obſervations on the inoculation of theſe children, and was aſtoniſhed at the ſlightneſs of the diſeaſe. He was particularly ſtruck with the perfect likeneſs of the puſtules with your beautiful copper-plates. He wiſhed to inform himſelf upon the ſubject of the Vaccine Inoculation, and ſoon convinced himſelf ſo fully of the importance of the diſcovery, that the next Sunday he gave from the pulpit the hiſtory of the Cow-pox Inoculation, and its immediate advantages; and concluded, by a very paternal and ſenſible exhortation to his pariſhioners, to avail themſelves of the benefit of it. He adopted this meaſure that he might aſcertain the confidence they had in his directions, and their affection for their children. His harangue had ſuch a happy effect, that the next Tueſday, the day fixed for my viſit to the village, I found thirty-eight children waiting for inoculation; and in three ſubſequent viſits I have inoculated more than eighty perſons. The diſtance of that place from Vienna not allowing me to go there ſo often as I wiſhed, the phyſician of the place, Dr. Ibezer, continues the Vaccine Inoculation, which has ſo much charmed all thoſe country people, that they come now to him without further exhortations, either of the curate or of the phyſician. If that example, of which I have given as much publicly as I could, ſhould be followed by the brethren of the worthy curate of Brunnam, how many thouſands lives, ſo precious to the country after a deſtructive war would be preſerved!

A young nobleman, Count Francon Hugues de Salm, diſtinguiſhes himſelf particularly by the encouragement which he gives to the Vaccine Inoculation at Brun, the capital of Moravia. He came on purpoſe to Vienna, to be acquainted by me of the beſt ſteps he had to take to introduce it. He examined ſome vaccine patients, to know what the vaccine puſtule ought to be. He has read all the Treatiſes he could collect on the ſubject. He has addreſſed an exhortation to the people of Moravia,

* We underſtand that this learned Phyſician is about to publiſh, in French and German, an elaborate Hiſtory of this Inoculation, dedicated to Lord Minto, as Miniſter of the nation where the diſcovery took place.

Moravia, to profit by your valuable difcovery. He has offered two prizes for the two Moravian phyficians who fhall inoculate the greateft number of children in the courfe of the year.* He has engaged intelligent phyficians to affift in thefe inoculations in a place of Prince Salm, his father's houfe, devoted to that purpofe: And, what is more curious, he has written a very good hiftory of the Vaccine difcovery, which he has diftributed among all the curates and fchoolmafters of Bohemia and Moravia. Thefe affociations are under my direction; they fend me their regifters, have their vaccine matter from me, &c. — Such patriotifm is truly praife-worthy, and reflects the greateft honour upon the young nobleman who has conceived and erected fo humane an inftitution.

I have introduced the Vaccine Inoculation in the Venetian State, under the direction of a very intelligent phyfician, Doctor Morefchi, who has begun it with the greateft fuccefs. I look upon Dr. M. to be perfectly well verfed in the new mode of inoculation, which I am at prefent far from confidering as a fubject to be committed to the hands of the inexperienced.

As Dr. Morefchi has begun his inoculations at Venice with the children of people of the firft rank, there is no doubt that their example will be followed very rapidly, and that Italy will foon enjoy the benefit of your difcovery.

The German phyficians are fo perfectly convinced of the advantages of this inoculation, that they have printed a fhort and popular exhortation to the people, which has been put into the hands of all clergymen, who are ordered to deliver a copy of it to the father and godfather of every child who is brought to church to be chriftened.

But among the various progreffes of the Cow-pox inoculation to which I have been inftrumental on the continent, none feem to me more interefting than its introduction at Conftantinople, under the aufpices of Lord Elgin, the Englifh Ambaffador, who difcharges by thefe means, in fome refpect, the debt which England and all Europe had contracted with the Turks for the inoculation of the Small-pox. His lordfhip had heard fuch favourable accounts of the Cow-pox, that he introduced it at Conftantinople by the inoculation of his own child, Lord Bruce.

[ The above is a very concife Extract from a long letter, in which the eulogiums on Doctor JENNER, and the importance of his difcovery, are omitted. ]

---

* Who has done this in England? The reward however of this difcovery, we hope, is delayed only to be increafed.

